# “Chronic fatigue, quality of life and long-term side-effects of chemotherapy in patients treated for non-epithelial ovarian cancer: national case-control protocol study of the GINECO-Vivrovaire rare tumors INCa French network for rare malignant ovarian tumors”

**DOI:** 10.1186/s12885-021-08864-8

**Published:** 2021-10-26

**Authors:** François Gernier, Djihane Ahmed-Lecheheb, Patricia Pautier, Anne Floquet, Cédric Nadeau, Sophie Frank, Jérôme Alexandre, Frédéric Selle, Dominique Berton-Rigaud, Elsa Kalbacher, Hubert Orfeuvre, Alain Lortholary, Paule Augereau, Fabien Labombarda, Lionel Perrier, Jean-Michel Grellard, Idlir Licaj, Bénédicte Clarisse, Aude-Marie Savoye, Héloise Bourien, Thibaut De La Motte Rouge, Jean-Emmanuel Kurtz, Katia Kerdja, Anaïs Lelaidier, Amandine Charreton, Isabelle Ray-Coquard, Florence Joly

**Affiliations:** 1 Clinical Research Department, Baclesse Cancer Center, 3 av. general Harris, 14076 Caen, France; 2 INSERM, U1086, Caen, France; 3 Gustave Roussy Cancer Center, Department of Medical Oncology, Université Paris-Saclay, Villejuif, France; 4 Bergonié Cancer Center, Bordeaux, France; 5 University Hospital Poitiers, Poitiers, France; 6 Curie Cancer Center, Paris, France; 7 University Hospital Cochin Hôtel-Dieu Broca, Paris, France; 8 Hospital Diaconesses-Croix St Simon, Paris, France; 9 Integrated Center for Oncology Nantes-Angers, Saint Herblain, France; 10 University Hospital Jean Minjoz, Besançon, France; 11 Hospital Fleyriat, Bourg-En-Bresse, France; 12 Catherine de Sienne Center, Nantes, France; 13 Integrated Center for Oncology Nantes-Angers, Angers, France; 14 Department of Cardiology, University Hospital Caen, Caen, France; 15 University of Lyon, Centre Léon Bérard, GATE L-SE UMR 5824, Lyon, France; 16 Jean Godinot Cancer Center, Reims, France; 17 Eugène Marquis Cancer Center, Rennes, France; 18 University Hospital Strasbourg, Strasbourg, France; 19 North-West Canceropole Data Center, Baclesse Cancer Center, Caen, France; 20 Léon Bérard Cancer Center, Université Claude Bernard, laboratoire HESPER, Lyon, France; 21 Université de Caen Basse-Normandie, UMR-S1077, Caen, France; 22 Department of Oncology, CHU de Caen, Caen, France

**Keywords:** Germ cell ovarian neoplasms, Sex cord stromal tumors, Long-term effects, Survivorship, Chemotherapy, Fatigue, Quality of life, Physical sequelae, Cardiovascular and pulmonary disorders

## Abstract

**Background:**

Germ cell tumors and sex cord stromal tumors are rare cancers of the ovary. They mainly affect young women and are associated with a high survival rate. The standard treatment mainly involves conservative surgery combined with chemotherapy [bleomycin, etoposide and cisplatin (BEP)] depending on the stage and the prognostic factors, as for testicular cancers. As reported in testicular cancer survivors, chemotherapy may induce sequelae impacting quality of life, which has not yet been evaluated in survivors of germ cell tumors and sex cord stromal tumors. The GINECO-VIVROVAIRE-Rare tumor study is a two-step investigation aiming to assess i) chronic fatigue and quality of life and ii) long-term side-effects of chemotherapy with a focus on cardiovascular and pulmonary disorders.

**Methods:**

Using self-reported questionnaires, chronic fatigue and quality of life are compared between 134 ovarian cancer survivors (cancer-free ≥2 years after treatment) treated with surgery and chemotherapy and 2 control groups (67 ovarian cancer survivors treated with surgery alone and 67 age-matched healthy women). Medical data are collected from patient records. In the second step evaluating the long-term side-effects of chemotherapy, a subgroup of 90 patients treated with chemotherapy and 45 controls undergo the following work-up: cardiovascular evaluation (clinical examination, non-invasive cardiovascular tests to explore heart disease, blood tests), pulmonary function testing, audiogram, metabolic and hormonal blood tests. Costs of sequelae will be also assessed. Patients are selected from the registry of the INCa French Network for Rare Malignant Ovarian Tumors, and healthy women by the ‘Seintinelles’ connected network (collaborative research platform).

**Discussion:**

This study will provide important data on the potential long-term physical side-effects of chemotherapy in survivors of Germ Cell Tumors (GCT) and Sex Cord Stromal Tumors (SCST), especially cardiovascular and pulmonary disorders, and neurotoxicity. The identification of long-term side-effects can contribute to adjusting the treatment of ovarian GCT or SCST patients and to managing follow-up with adapted recommendations regarding practices and chemotherapy regimens, in order to reduce toxicity while maintaining efficacy. Based on the results, intervention strategies could be proposed to improve the management of these patients during their treatment and in the long term.

**Trial registration:**

This trial was registered at clinicaltrials.gov: 03418844, on 1 February 2018.

This trial was registered on 25 October 2017 under the unique European identification number (ID-RCB): 2017-A03028–45.

Recruitment Status: Recruiting.

**Protocol version:**

Version n° 4.2 dated from Feb 19, 2021.

**Trial sponsor:**

Centre François Baclesse, 3 avenue du Général Harris, F-14076 Caen cedex 05, France.

## Background

Malignant non-epithelial ovarian tumors are rare cancers that account for less than 20% of ovarian cancers in adults [1]. The main ones are Germ Cell Tumors (GCT) and Sex Cord Stromal Tumors (SCST). They mainly affect young women, are diagnosed early and have a good prognosis and long survival. GCT mainly affects teenagers and young women between 15 and 30 years. They have a good prognosis whatever the stage, with a 10-year survival rate up to 81% [2]. Initial treatment includes conservative surgery (with fertility-sparing for young women) combined with adjuvant chemotherapy [bleomycin, etoposide, and platinum (BEP)], depending on the stage and prognostic factors, as in chemotherapy for testicular cancer [3]. The choice of the optimal chemotherapy regimen for ovarian GCT has been based on standards for testicular cancer. SCST are also rare tumors that occur at any age with a peak incidence between 20 and 40 years. In 70% of cases, they are diagnosed early and have a high rate of remission, with an overall 5-year survival rate of 85% [4]. The main treatment of SCST is also conservative surgery for young women, depending on the tumor extension, associated with the same chemotherapy regimen as for GCT tumors (i.e. BEP) for extensive or recurrent disease.

While follow-up in GCT and SCST ovarian survivors over several years focuses on the risk of recurrence, there is no consensus on follow-up modalities in patients who relapse. Furthermore, the late effects of chemotherapy (metabolic, cardiac, respiratory, renal, hematological disorders, ototoxicity and neurotoxicity) are not routinely investigated. However, follow-up of testicular cancer survivors treated with the same chemotherapy found persistent long-term side-effects of the chemotherapy such as chronic fatigue, cardiovascular and pulmonary disease, neurotoxicity, hypogonadism and a higher risk of secondary cancer. Cisplatin and bleomycin induce alterations in endothelial function and endothelial damage that may trigger vascular diseases [5]. After platinum-based chemotherapy, testicular cancer survivors have a 2-to-3-fold greater risk of cardiovascular disease compared with patients treated with surgery alone or individuals in the general population [6–9]. Raynaud’s syndrome is also a frequent occurrence, with a 2-to-4-fold increased risk after receiving a high platinum dose [10]. In addition, these patients often present a metabolic syndrome, which is a strong predictor of cardiovascular diseases. Metabolic syndrome occurs in 20–30% of long-term testicular cancer patients, and onset is much earlier (3–5 years after treatment) than would be expected in the general population [11, 12]. Other toxicities such as pulmonary toxicity, renal toxicity, ototoxicity and neurological sequelae are frequent and dose-related [13]. According to the available data, the relative risk of a second cancer is approximately doubled after chemotherapy. The estimated cumulative risk of leukemia among testicular cancer survivors who are given etoposide at total doses of less than or equal to 2000 or more than 2000 mg/m2 is 0.5 and 2%, respectively [14].

Testicular cancer survivors have a 6% increased risk of dying of non-cancer causes (infections, cardiovascular disease) after cisplatin-based chemotherapy compared with the general population [15]. Long-term toxicity has been associated with an increased risk of mortality due to pulmonary diseases [16]. Furthermore, the physical effects of chemotherapy and factors associated with the disease such as stress, anxiety and depression have an impact on the quality of life (QoL) of testicular cancer survivors in the physical, psychological, sexual and social domains [17, 18]. Fatigue has been described as one of the most distressing adverse effects of cancer and its treatment. A statistically significant higher frequency of chronic cancer-related fatigue (duration of > 6 months) among long-term testicular cancer survivors (17%) compared with men in the general population (10%) (*P* < .001) has been reported [19]. In the long term, chronic fatigue is strongly associated with poor QoL and numerous psychological and somatic problems. In a longitudinal study exploring chronic fatigue in 812 testicular cancer survivors, prevalence of chronic fatigue increased significantly over time. After 19 years of follow-up, 27% of patients reported fatigue and the risk of chronic fatigue was increased 3-to-4-fold in patients with high levels of neuropathy compared with no neuropathy, 2-to-3-fold for high levels of Raynaud-like phenomena, and 2-to-4-fold for higher levels of anxiety and depression [20]. The late effects of BEP experienced by testicular cancer survivors such as cardiovascular disorders may occur as early as the first year post-treatment [8].

While these issues have received considerable attention in testicular cancer survivors, it is not the case for women treated for non-epithelial ovarian cancers. We hypothesize that the same or similar difficulties and late effects of chemotherapy are also experienced by survivors of ovarian GCT and SCST. In turn, this would have a late impact on their general health, QoL and social and professional integration, as already demonstrated in testicular cancer survivors. The impact of treatment on hormonal status, with consequences on fertility, menopausal status and sexuality, is also crucial in young patients treated for rare ovarian cancer [21, 22]. To our knowledge, very few studies have focused on the impact of chemotherapy on the general health and different domains of QoL of ovarian GCT and CSCT survivors. In a previous study, patients reported significantly greater reproductive concerns and less sexual pleasure than controls. They also more often reported chemotherapy-related effects such as hypertension, hypercholesterolemia and hearing loss [23, 24]. The rate of pulmonary toxicity was greater in SCST patients treated with chemotherapy containing platinum and bleomycin (incidence rate = 7.7% and mortality = 1.8%) [25]. Moreover, in a recent study, hearing disorders were observed in 22% of patients treated with cisplatin-based chemotherapy versus 15% in healthy subjects of the same age [26]. The real impact of chemotherapy on accumulated long-term toxicities and the impact on the different domains of QoL in ovarian GCT and CSCT survivors have not been assessed, and studies to date have been based only on self-reported questionnaires, none of which specifically investigated cardiovascular and pulmonary disorders.

The identification of the long-term side-effect and the impact on QoL encountered by these survivors is thus a prerequisite for proposing and assessing intervention strategies to improve the management of these patients during treatment and over the long term.

## Methods/design

### Objectives

We propose to conduct a large multidisciplinary multicenter case-control study using the INCa French network for rare malignant ovarian tumors TMRG (Tumeurs Malignes Rares Gynecologiques) [27]. The study will explore the needs and difficulties encountered by ovarian GCT and SCST survivors after treatment with surgery (with or without fertility-sparing) and chemotherapy in their daily life, and identify the late effects of chemotherapy.

This case-control study will be conducted in two phases to assess the following:

i) chronic fatigue and several domains of QoL; and
ii) the long-term medical side-effects of chemotherapy with a focus on cardiovascular and pulmonary disorders, and neurotoxicity.

#### Step 1:

##### Primary objective

The main objective is to assess chronic fatigue in survivors treated for ovarian GCT or SCST with surgery and chemotherapy compared with patients treated with surgery alone and with age-matched healthy women (± 2 years).

The **secondary objectives** are to assess:

- Fertility follow-up and parental projects according to age (≤ 45 years);
- Menopausal symptoms and their impact on QoL;
- The impact of cancer and its treatments on personal trajectory and professional status (access to work, professional ambition, financial situation, etc.);
- The different dimensions of QoL including health-related QoL (anxiety, depression, fear of recurrence and sexuality), sleep disturbance, physical activity and living conditions [28] (relationship with partner, family and entourage, consumption of drugs, use of healthcare institutions and social support);
- Self-reported neurotoxicity and cognitive impairment.

#### Step 2:

##### Primary objective

The main objective is to assess the late clinical effects of chemotherapy with a focus on cardiovascular and pulmonary disorders.

##### Secondary objectives

Metabolic and hormonal disorders, neurotoxicity and ototoxicity, second cancer, Raynaud’s syndrome, and costs of sequelae are also assessed.

##### Design and setting of the study

**Fig. 1:**
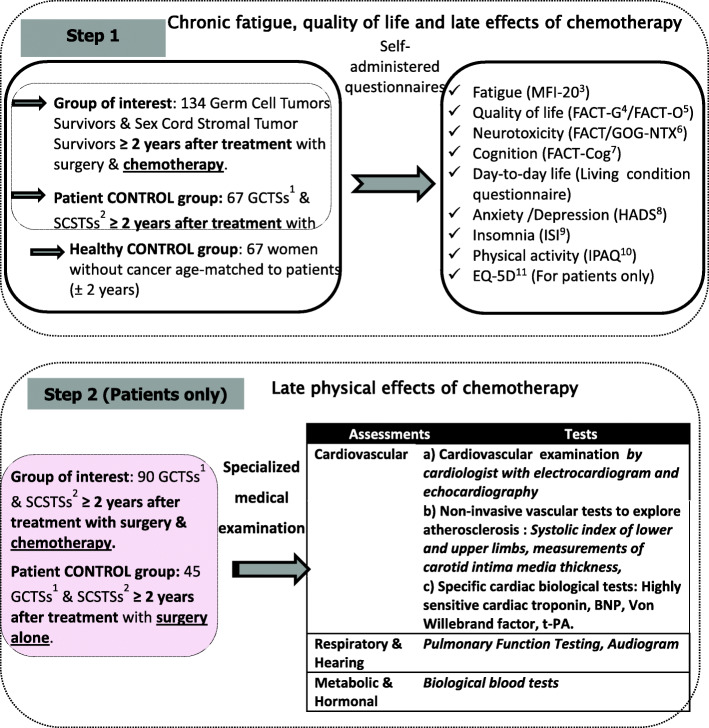
STUDY Design. ^1^ GCTSs: Germ Cell Tumor Survivors; ^2^ SCSTSs: Sex Cord Stromal Tumor Survivors; ^3^MFI: Multidimensional Fatigue Inventory; ^4^ FACT-G: General Functional Assessment of Cancer Therapy; ^5^FACT-O: Functional Assessment of Chronic Therapy- Ovarian subscale,^6^ FACT/GOG-Ntx: Functional Assessment of Cancer Therapy/ Gynecologic Oncology Group–Neurotoxicity subscale; ^7^ FACT-Cog: Functional Assessment of Cancer Therapy-Cognitive; ^8^ HADS: Hospital Anxiety and Depression Scale,^9^ ISI: Insomnia Severity Index,^10^ IPAQ: International Physical Activity Questionnaire;^11^ EQ-5D Euroqol questionnaire

This is a large two-phase multicenter case-control study (Fig. 1). The study protocol and this manuscript have been written in accordance with standard protocol items, namely recommendations for interventional trials (SPIRIT).

### Study participants

**134 adult ovarian GCT or SCST survivors in remission ≥ 2 years after treatment** with **surgery and BEP chemotherapy** (≥ 1 cycle) (recurrence occurs mainly in first 2 years after initial treatment) are compared to **two groups of controls**:

1. **Patient control group:** 67 adult ovarian GCT or SCST survivors in remission ≥2 years after treatment with surgery alone;
2. **Healthy control group:** 67 age-matched (±2 years) healthy women without cancer and without serious chronic diseases.

Eligible patients of interest and control groups are recruited from the INCa French network TMRG. These patients have been selected by the oncologists of the French cooperative GINECO group (Groupe d’Investigateurs Nationaux pour l’Étude des Cancers Ovariens et du sein). There are 19 participating centers. Healthy women age-matched with patients of interest are recruited from the Seintinelles® network (https://www.seintinelles.com/home). Seintinelles® is a collaborative research platform linking researchers and citizens to accelerate cancer research. Female cancer-free volunteers will be contacted to complete online self-questionnaires. Inclusion and exclusion criteria are detailed in Table 1. A coordinating committee was set up before the conception of the research including the scientific team of TMRG and GINECO and cardiologists.

**Table 1:** Study inclusion and exclusion criteria

	Group of interest	Patient control group	Healthy control group
Step 1	**134** ovarian Germ Cell Tumor or Sex Cord Stromal tumor survivors	**67** ovarian Germ Cell Tumor or Sex Cord Stromal tumor survivors	**67** healthy women
Step 2	**90** ovarian Germ Cell Tumor or Sex Cord Stromal tumors survivors	**45** ovarian Germ Cell Tumor or Sex Cord Stromal tumor survivors	**Not applicable**^**a**^
Inclusion criteria	≥ 18 years old	≥ 18 years old	≥ 18 years old
		Age-matched to group of interest (± 2 years)	Age-matched to group of interest (± 2 years)
	Patient treated with **surgery** and **BEP chemotherapy** (≥ 1 cycle)	Patient treated with **surgery alone** (with or without fertility sparing).	
	Patient in remission ≥2 years after initial treatment	Patient in remission ≥2 years after initial treatment	
	Recurrence authorized if remission more than 2 years after end of initial treatment	Recurrence authorized if remission more than 2 years after the end of initial treatment	
	Patient with no other cancers (except basal cell skin carcinoma, breast cancer and cervical cancer)	Patient with no other cancers (except basal cell skin carcinoma, breast cancer and cervical cancer)	Women without cancer or serious chronic diseases
	Patient having signed consent to participate	Patient having signed consent to participate	Healthy women accepted to complete online self-reported questionnaires
Exclusion criteria	Pregnant or breastfeeding woman		
	Psychiatric disorders		
	Major subject to legal protection or unable to express consent		

*BEP* Bleomycin, Etoposide and Cisplatinum

^a^: Healthy control group participated only in step 1 of study (self-reported questionnaire)

#### Study sites.

The list of study sites is available on https://clinicaltrials.gov/ct2/show/NCT03418844 .

### Assessments

- **Step 1**

All participants are asked to complete several validated self-reported questionnaires including standardized and validated questionnaires (Table 2):

- **Fatigue Questionnaire (Multidimensional Fatigue Inventory: MFI-20)**: includes 20 items measuring 5 dimensions of fatigue (general and physical perception of fatigue, reductions in motivation and activity, and mental fatigue) [29, 30].
- **Modified Living Conditions Questionnaire**: with objective questions on fertility monitoring and parental plans, social reintegration, career path and professional situation, care consumption and patient expectations regarding post-cancer management. This questionnaire used in previous surveys [28–31].
- **Functional assessment of cancer therapy scale general** (FACT-G) and module for **ovarian cancer** (FACT-O): symptomatic scale comprising 12 items assessing abdominal symptoms, other adverse effects of chemotherapy, hormonal disorders, body image, sexuality and attitude towards the disease and its treatments [32].
- **Hospital Anxiety and Depression Scale** (HADS): A 14-item structured self-administered questionnaire to screen for anxiety and depression. This scale has been validated in oncology [33].
- **Peripheral Neurotoxicity Questionnaire** (FACT/GOG-NTX): specific scale composed of 11 items probing the following points: numbness or tingling, discomfort, arthralgia, cramps, general weakness, tinnitus, hearing problems, problems buttoning clothes, deep sensitivity problems (touching small objects), walking problems.
- **FACT-Cog Questionnaire**: A self-questionnaire that subjectively assesses patients’ cognitive complaints. Patients are asked to rate their feelings on a scale ranging from 0 “not at all” to 4 “very much”. The 37 items cover 4 dimensions: perception of cognitive impairment, impact on QoL, comments from third parties, perception of cognitive abilities [34].
- **Insomnia Severity Index (ISI)**: consists of 7 items used to quantitatively assess how much the person is affected by insomnia [35].
- **Physical Activity Questionnaire** (IPAQ): has the advantage of providing a measure of overall physical activity as well as its frequency and intensity.
- **Self-administered Euroqol Questionnaire** (EQ-5D 5 L): allows the evaluation of a utility score associated with a health condition. The value set based on societal preferences of the French population recently published will be used [36].

**Table 2:** Self-reported questionnaires used in study

Questionnaires	Scoring	Definition of scoring
**Chronic fatigue**		
**Fatigue** MFI-20 items	5 dimensions of fatigue: -General fatigue -Physical fatigue -Reduced motivation -Reduced activity -Mental fatigue	High score indicates high level of fatigue
**Global quality of life**^**a**^		
**Global QoL** FACT-G	4 dimensions: 0–4 Likert scale - PWB: 7 items, score range: 0–28 - SWB: 7 items, score range: 0–28 - EWB: 6 items, score range: 0–24 - FWB: 7 items, score range: 0–28 Total score: 0–108	A lower score indicates a lower QoL dimension
**Additional concerns subscale**^**a**^		
**EOC specific concerns** FACT-O	0–4 Likert scale Score: 0–44	A lower score indicates a severe symptom
		A lower score indicates a high level of fatigue
**Neurotoxicity** FACT/GOG-Ntx	0–4 Likert scale Score range: 0–44	A lower score indicates a worse neuropathy Score < 33 ≈ Severe neuropathy
**TOI = PWB + FWB + concerns subscale**^**a**^		
FACT-F/TOI	Score range: 0–108	A lower score indicates a severe symptom
FACT/GOG-Ntx/TOI	Score range: 0–100	
FACT-Cog	Score range: 0–100	
FACT-O/ TOI	Score range: 0–100	
**Other dimensions of QoL**		
**Anxiety & Depression** HADS	Anxiety: 7 items, score range: 0–21 Depression:7 items, score range: 0–21	Score ≥ 8 ≈ elevated anxiety, Score ≥ 8 ≈ elevated depression,
**Sleep disturbance** ISI	0–5 Likert scale Score range: 0–28	A higher total score indicates more severe sleep difficulties Score 22–28 ≈ Severe clinical insomnia
**Physical activity** IPAQ	3 levels of physical activity (categorical score)^b^	High/ moderate/low level of physical activity
**Conditions of life**		
**Living conditions** questionnaire [25]	Daily life of participants (family, social and professional situation)	NA
**Assessment and comparison of the costs of sequelae in the 2 groups of patients**		
Euroqol (EQ-5D 5 L) questionnaire	Generic measure for clinical and economic assessment	Utility score associated with state of health.

^a^: Clinically significant difference: variation of 5%, ^b^: Responses are categorized into three levels of physical activity: low (<moderate); moderate (3 or more days/week of vigorous activity of at least 20 min/day or 5 or more days/week of moderate activity, or 30 min/day walking) and high (vigorous activity on at least 3 days or 7 days/week of any combination of walking, moderate or vigorous intensity activities). *MFI* Multidimensional Fatigue Inventory -*QoL* Quality of Life, *FACT-G* General Functional Assessment of Cancer Therapy, *PWB* Physical Well-Being, *SWB* Social/family Well-Being, *EWB* Emotional Well-Being, *FWB* Functional Well-Being, *EOC* Epithelial Ovarian Cancer, *FACT-O* Functional Assessment of Chronic Therapy- Ovarian subscale, *FACT-F* Functional Assessment of Chronic Therapy-Fatigue subscale, *FACT/GOG-Ntx* Functional Assessment of Cancer Therapy/ Gynecologic Oncology Group–Neurotoxicity subscale, *FACT-Cog* Functional Assessment of Cancer Therapy-Cognitive, *TOI* Trial Outcome Index, *ISI* Insomnia Severity Index, *HADS* Hospital Anxiety and Depression Scale, *IPAQ* International Physical Activity Questionnaire, *NA* Not Adapted

Volunteers from both patient groups receive information sheets and the different questionnaires from their oncologists during the follow-up consultation or by mail. They are asked to return completed questionnaires anonymously in a stamped preaddressed envelope. A reminder is sent if necessary.

**Patient’s medical data** (date and context of disease diagnosis, treatment modalities, fertility-sparing, second cancer, and comorbidities (focus on cardiovascular diseases, pulmonary and metabolic disorders)) are collected from patient records. For the healthy control group, the website administrator of the Seintinelles network publishes the study information and the questionnaires on their website, and contacts registered healthy women to complete the different questionnaires online.

- **Step 2**

The study (objectives and constraints) is proposed to both patient groups (90 patients with surgery and BEP chemotherapy and 45 with surgery alone) by GINECO oncologists. Once signed informed consent has been obtained, patients undergo cardiovascular, respiratory, hearing, metabolic and hormonal work-up. The planned tests are detailed in Table 3.

**Table 3:** Overview of study assessments of the VIVROVAIRE TR Study

	Study Period		
	Enrollment	Step 1	Step 2
**Eligibility Screen**	**•**		
**Informed Consent**	**•**		
**Disease medical history**	**•**		
**Chronic fatigue, quality of life and late effects of chemotherapy**		**•**	
✓ Fatigue (MFI-20^a^)		**•**	
✓ Quality of life (FACT-G^b^/FACT-O^c^)		**•**	
✓ Neurotoxicity (FACT/GOG-NTX^d^)		**•**	
✓ Cognition (FACT-Cog^e^)		**•**	
✓ Day-to-day life (Living condition questionnaire)		**•**	
✓ Anxiety /Depression (HADS^f^)		**•**	
✓ Insomnia (ISI^g^)		**•**	
✓ Physical activity (IPAQ^h^)		**•**	
✓ EQ-5D^i^ (For patients only)		**•**	
**Specialized medical examination**			**•**
**Cardiovascular:**			
a) Cardio-vascular medical examination with non-invasive tests to explore heart disease (by a cardiologist)			**•**
- Electrocardiogram			**•**
- Echocardiography			**•**
-Coronary endothelium-dependent vasoreactivity testing (optional exam)			**•**
b) Non-invasive vascular tests to explore atherosclerosis:			**•**
- Systolic index of lower and upper limbs,			**•**
-Ultrasound images to measure carotid intima media thickness			**•**
- Capillaroscopy to assess Raynaud’s syndrome (optional exam)			**•**
c) Specific cardiac biological tests:			**•**
High-sensitive cardiac troponin, BNP, Von Willebrand factor (VWf), tissular Plasminogen Activator (t-PA).			**•**
**Respiratory & Hearing:**			**•**
- Respiratory Function Tests (RFT)			**•**
- Audiogram			**•**
**Blood sampling: exploring metabolic and hormonal disorders**			**•**
- Hormonal assessment: sex hormone binding globulin (SHBG), Luteinizing hormone (LH), Follicle-stimulating hormone (FSH), Estradiol, Anti-Müllerian hormone (AMH), thyroid stimulating hormone (TSH).			**•**
-Carbohydrate-lipid balance: fasting blood glucose and insulin, lipid fractions, triglycerides;			**•**
- Osteocalcin blood Tests: calcium, phosphorus, vitamin D			**•**
- Renal assessment: ionograms, creatinine level			**•**
-Hepatic assessment: transaminases (TGO, TGP), Aspartate Aminotransferase (ASAT), Alanine Aminotransferase (ALAT), Alkaline Phosphatase (PAL), Gamma-glutamyl transferase (GGT), Lactate dehydrogenase (LDH			**•**
- C-Reactive Protein Test ultrasensitive			**•**

^a^*MFI* Multidimensional Fatigue Inventory;^b^*FACT-G* General Functional Assessment of Cancer Therapy; ^c^*FACT-O* Functional Assessment of Chronic Therapy- Ovarian subscale,^d^*FACT/GOG-Ntx* Functional Assessment of Cancer Therapy/ Gynecologic Oncology Group–Neurotoxicity subscale; ^e^*FACT-Cog* Functional Assessment of Cancer Therapy-Cognitive;^f^*HADS* Hospital Anxiety and Depression Scale,^g^*ISI* Insomnia Severity Index,^h^*IPAQ* International Physical Activity Questionnaire; ^i^EQ-5D Euroqol questionnaire

Patients who have agreed to participate in the second stage will undergo the following medical check-up:

**Cardiac check-up** which will include:

- A cardiac consultation including the measurement of systolic pressure index;
- An electrocardiogram;
- Carotid Doppler ultrasound with measurement of carotid intima media thickness and arterial; vasoreactivity (arterial elasticity, carotid pulse wave velocity) (optional for vasoreactivity),
- A capillaroscopy: search for Raynaud’s syndrome (optional);
- A humeral Doppler ultrasound for study of flow-mediated humeral vasodilatation (optional);
- A trans-thoracic echocardiography 2D, 2D Strain (optional) and ± 3D;
- A blood test comprising an enzyme profile: ultra-sensitive troponin, BNP, von Willebrand factor assay, and t-PA.

### Lung and hearing examination

- Respiratory Function Tests (RFT);
- Tonal audiogram.

**Blood sampling**: exploring metabolic and hormonal disorders.

- Carbohydrate-lipid balance: fasting blood glucose and insulin, lipid fractions, triglycerides;
- Hepatic: transaminases (TGO, TGP), Aspartate Aminotransferase (ASAT), Alanine Aminotransferase (ALAT), Alkaline Phosphatase (PAL), Gamma-glutamyl transferase (GGT), Lactate dehydrogenase (LDH);
- Hormone balance: sex hormone binding globulin (SHBG), Luteinizing hormone (LH), Follicle-stimulating hormone (FSH), Estradiol, Anti-Müllerian hormone (AMH), thyroid stimulating hormone (TSH).
- Osteocalcic balance: calcium, phosphorus, vitamin D;
- Renal assessment: ionogram, creatinine;
- C-reactive protein (CRP) + highly sensitive CRP.

Compensation is offered to cover the costs of transport and to compensate one day off work.

The overview of study assessments and procedures can be found in Table 3.

**The costs of the sequelae** will be estimated for the 140 patients. Standard unit costs will be calculated for each type of sequelae using data from the financial departments of the participating hospitals, as well as the published literature [37]. The average costs will be assessed and compare between both patient groups. One-way sensitivity analyses will be conducted by varying parameters by plus or minus 20% and illustrated graphically in a Tornado diagram.

### Statistical analysis

#### Sample size

- **Step 1 = 268 participants**

The first aim is to show a difference in the proportion of patients with chronic fatigue (≥1 dimension of MFI-20) in the group of interest as compared with each of the control groups. The pairwise comparison will be performed using the χ^2^ test (one-tailed test under the assumption of higher chronic fatigue in the chemotherapy group of interest) at a risk α = 0.05 and a power level of 80% (1-β = 80%). Assuming that 25% of patients express chronic fatigue in the group of interest as described in testicular cancer [20] and 10% in each of the control group, the required sample size, with a 2:1 allocation ratio in favor of the group of interest, is 121 subjects in the group of interest and 61 subjects in each of the two control groups (patient control and healthy control).. To anticipate 10% of non-assessable women, we plan to enroll 134 survivors in the group of interest, 67 survivors in the control group, and 67 healthy controls.

- **Step 2 = 135 patients**

Assuming around 66% of patients will accept to participate, 90 patients in the group of interest and 45 patients in patient control group are expected to agree to participate.

Assuming 10% of non-assessable participants (around 80 and 40 survivors in the group of interest and control group, respectively), and a proportion of patients experiencing cardiac sequelae varying from 5 to 35% (with a 95% interval confidence) according to literature for testicular cancer [38], it will be possible to estimate the frequency of cardiac sequelae with a precision of 4.8 to 10.5%, in the group of interest and 6.8 to 14.8% in the control group.

### Data management

A Web Based Data management system (Ennov Clinical (version 7.5.10, ENNOV / CLINSIGHT, 33155 Cenon, France)) will be used for data collection and query handling. The investigator will ensure that data are recorded on the electronic case report form CRFs as specified in the study protocol and in accordance with the instructions provided. The data will have to be filled in in these eCRF as they are obtained, and the sponsor will take charge of the monitoring.

The investigator ensures the accuracy, completeness, and timeliness of the data recorded and of the provision of answers to data queries according to the Clinical Study Agreement. A copy of the completed CRFs will be archived at the study site.

All data will be handled and stored according to the EU General Data Protection Regulation (GDPR).

#### Planned analysis

Data analyses will be conducted according to the statistical methods used in paired case-control studies, as follows:

1. Descriptive analysis of patients’ and controls’ participation and according to sociodemographic and clinical data (including tumor type), quantitative QoL scores and data on living conditions in patients and controls, and then in patients based on medical data related to cancer.
2. Statistical comparison using a univariate and multivariate analysis (ANOVA, or Kruskal-Wallis test, or Mc Nemar χ2 test depending on the nature of the variables and GLM (Generalized Linear Model) model with a Bonferroni correction):

- Socio-demographic features and comorbidities;
- Reintegration and sequelae data, then by subgroup (including fertility-sparing);

A prognostic model for predicting QoL or living conditions has been constructed using an adapted model (GLM), taking into account the time since end of treatment and controlling for effect of center.

Medical and biological parameters are the categorical variables. They will be compared between the two groups of patients and then between sub-groups according to time since end of treatment (short term and long-term follow-up) using the χ2 test or the Fisher exact test.

Costs will be compared using the Wilcoxon-Mann-Whitney test. Standard multivariate analyses will be implemented in order to control the potential selection bias. Uncertainties regarding costs of the sequelae will be assessed by probabilistic analysis using nonparametric bootstrap methods: 1000 simulated bootstrap samples will be generated by independent draws. All 95% confidence intervals will be computed. All analyses will be performed using STATA software version 16.0 (StataCorp, College Station, TX).

## Discussion

Regarding the late effects of chemotherapy in testicular cancer survivors, some of which occur as early as in the first year after treatment, and the lack of data in ovarian GCT and SCST survivors, research is needed on these issues in rare ovarian cancer survivors, particularly germ cell and sex cord stromal tumors, i.e. the main non-epithelial ovarian cancers.

To our knowledge, this is the first large multicenter case-control study to assess chronic fatigue, several domains of QoL and to explore the late effects of chemotherapy, particularly cardiovascular and pulmonary disorders and neurotoxicity. The study is based on the INCa French Network TMRG – GINECO and is representative of the French population treated for ovarian GCT and SCST. It offers the opportunity to better understand the impact of cancer and chemotherapy on long-term fatigue and quality of life in a large population of non-epithelial ovarian cancer survivors. The comparison with (i) patients treated with surgery alone and (ii) women without cancer will provide information about the needs and concerns of these patients at distance from treatment, i.e. follow-up of fertility, parental projects, social and professional integration, etc. The expected results will further understanding of the post-treatment period in survivors of rare cancer, especially young women.

The rigorous medical work-up conducted nationwide and focusing on the late effects of chemotherapy, with a focus on cardiovascular and pulmonary disorders, will provide important data about the physical and functional late effects of chemotherapy. As a result, intervention strategies could be proposed to improve the management of these patients during treatment and in the long term. In addition, specific strategies for post-treatment care and follow-up will become possible. These longitudinal evaluations will allow the late effects of cancer treatments to be measured and conclusions to be drawn.

In terms of public health, the detailed information that will be provided by this ambitious multidisciplinary research, involving oncologists, cardiologists, vascular physicians, otorhinolaryngologists and pneumologists, is the prerequisite for envisaging interventional strategies and treatments for these patients over time. The identification of sequelae would fuel recommendations regarding practices and chemotherapy regimens that reduce toxicity while maintaining efficacy. In the long term, the resulting preventive actions against the potential side-effects of chemotherapy identified in this research would have a beneficial impact on public health expenditure.

This original study will provide important data on the potential long-term physical side-effects of chemotherapy, especially cardiovascular and pulmonary disorders, neurotoxicity and the impact on quality of life. Based on the expected results, intervention strategies could be proposed to improve the management of these patients during their treatment and over time. By identifying the long-term side-effects of these chemotherapy regimens, it will be possible to produce recommendations aiming to reduce their toxicity while maintaining their efficacy.

### Trial status

Recruiting.

## Data Availability

The datasets used and/or analyzed during the current study are available from the corresponding author on reasonable request. Grant number: ARC 2016 PGA1 20160203900.

## Abbreviations

AMH: Anti-Müllerian hormone
BEP: Bleomycin, Etoposide and Cisplatin
CT: Chemotherapy
eCRF: electronic case report form
FSH: Follicle-stimulating hormone
GINECO: Groupe d’Investigateurs Nationaux pour l’Étude des Cancers Ovariens et du sein
GCT: Germ Cell Tumor
INCa: Institut National du Cancer
QoL: Quality of Life
SCST: Sex Cord Stromal Tumor
SHBG: Sex Hormone Binding Globulin
SPIRIT: Standard Protocol Items, namely Recommendations for Interventional Trials
TMRG: Tumeurs Malignes Rares Gynécologiques
t-PA: *tissular* Plasminogen Activator
TSH: Thyroid Stimulating Hormone
VWf: Von Willebrand factor

## References

[1] Bats AS, Larousserie F, Le Frère Belda MA, Metzger U, Lécuru F (2009). Update on malignant non epithelialovariantumours. GynecolObstetFertil..

[2] Trétarre B, Molinié F, Woronoff AS, Bossard N, Bessaoud F, Marrer E, Grosclaude P, Guizard AV, Delafosse P, Bara S, Velten M, Lapôtre-Ledoux B, Ligier K, Léone N, Arveux P, Uhry Z (2015). Ovarian cancer in France: trends in incidence, mortality and survival, 1980-2012. GynecolOncol..

[3] Schwartz PE, Chambers SK, Chambers JT, Kohorn E, Mc IS (1992). Ovarian germ cell malignancies: the Yale University experience. GynecolOncol.

[4] Ray-Coquard I, Brown J, Harter P, Provencher DM, Fong PC, Maenpaa J, Ledermann JA, Emons G, Rigaud DB, Glasspool RM, Mezzanzanica D, Colombo N (2014). Gynecologic Cancer InterGroup (GCIG) consensus review for ovarian sex cord stromal tumors. Int J Gynecol Cancer.

[5] Nuver J, de Haas EC, Van Zweeden M, Gietema JA, Meijer C (2010). Vascular damage in testicular cancer patients: a study on endothelial activation bybleomycin and cisplatin in vitro. Oncol Rep.

[6] Altena R, Hummel YM, Nuver J, Smit AJ, Lefrandt JD, de Boer RA, Voors AA, van den Berg MP, de Vries EGE, Boezen HM, Gietema JA (2011). Longitudinal changes in cardiac function after cisplatin-based chemotherapy for testicular cancer. Ann Oncol.

[7] Aziz NM (2007). Cancer survivorship research: state of knowledge, challenges and opportunities. ActaOncol.

[8] Altena R, de Haas EC, Nuver J, Brouwer CA, van den Berg MP, Smit AJ, Postma A, Sleijfer DT, Gietema JA (2009). Evaluation of sub-acute changes in cardiac function after cisplatin-based combination chemotherapy for testicular cancer. Br J Cancer.

[9] vanSchinkel LD, Willemse PM, van der Meer RW, Burggraaf J, van Elderen SG, Smit JW, de Roos A, Osanto S, Lamb HJ (2013). Chemotherapy for testicular cancer induces acute alterations in diastolic heart function. Br J Cancer.

[10] Sprauten M, Darrah TH, Peterson DR, Campbell ME, Hannigan RE, Cvancarova M, Beard C, Haugnes HS, Fosså SD, Oldenburg J, Travis LB (2012). Impact of long-term serum platinum concentrations on neuro- and ototoxicity in Cisplatin-treated survivors of testicular cancer. J ClinOncol..

[11] Willemse PM, Burggraaf J, Hamdy NA, Weijl NI, Vossen CY, van Wulften L, van Steijn-van Tol AQ, Rosendaal FR, Osanto S (2013). Prevalence of the metabolic syndrome and cardiovascular disease risk in chemotherapy-treated testicular germ cell tumour survivors. Br J Cancer.

[12] de Haas EC, Altena R, Boezen HM, Zwart N, Smit AJ, Bakker SJ, van Roon AM, Postma A, Wolffenbuttel BH, Hoekstra HJ, van Leeuwen FE, Sleijfer DT, Gietema JA (2013). Early development of the metabolic syndrome after chemotherapy for testicular cancer. Ann Oncol.

[13] Brydoy M, Oldenburg J, Klepp O, Bremnes RM, Wist EA, Wentzel-Larsen T, Hauge ER, Dahl O, Fossa SD (2009). Observational study of prevalence of long-term Raynaud-like phenomena and neurological side-effects in testicular cancer survivors. J Natl Cancer Inst.

[14] Travis LB, Beard C, Allan JM, Dahl AA, Feldman DR, Oldenburg J, Daugaard G, Kelly JL, Dolan ME, Hannigan R, Constine LS, Oeffinger KC, Okunieff P, Armstrong G, Wiljer D, Miller RC, Gietema JA, van Leeuwen FE, Williams JP, Nichols CR, Einhorn LH, Fossa SD (2010). Testicular cancer survivorship: research strategies and recommendations. J Natl Cancer Inst.

[15] Haugnes HS, Oldenburg J, Bremnes RM (2015). Pulmonary and cardiovascular toxicity in long-term testicular cancer survivors. UrolOncol..

[16] Fosså SD, Gilbert E, Dores GM, Chen J, McGlynn KA, Schonfeld S, Storm H, Hall P, Holowaty E, Andersen A, Joensuu H, Andersson M, Kaijser M, Gospodarowicz M, Cohen R, Pukkala E, Travis LB (2007). Noncancer causes of death in survivors of testicular cancer. Natl Cancer Inst.

[17] Vehling S, Mehnert A, Hartmann M, Oing C, Bokemeyer C, Oechsle K (2016). Anxiety and depression in long-term testicular germ cell tumor survivors. Gen Hosp Psychiatry.

[18] Alacacioglu A, Ulger E, Varol U, Yavuzsen T, Akyol M, Yildiz Y, Yildiz I, Bayoglu V, Dirican A, Demir L, Salman T, Kucukzeybek Y, Alacacioglu I, Can H, Tarhan MO (2014). Sexual satisfaction, anxiety, depression and quality of life in testicular cancer survivors. Med Oncol.

[19] Orre IJ, Fossa SD, Murison R (2008). Chronic cancer-related fatigue in long-term survivors of testicular cancer. J Psychosom Res.

[20] Sprauten M, Haugnes HS, Brydøy M, Kiserud C, Tandstad T, Bjøro T, Bjerner J, Fosså SD, Oldenburg J, Cvancarova M1 (2015). Chronic fatigue in 812 testicular cancer survivors during long-term follow-up: increasing prevalence and risk factors. Ann Oncol.

[21] Fischer OJ, Marguerie M, Brotto LA (2020). Sexual function, quality of life, and experiences of women with ovarian Cancer: a mixed-methods study. Sex Med.

[22] Ceppi L, Galli F, Lamanna M, Magni S, Dell'Orto F, Verri D, Delle Marchette M, Lissoni AA, Sina F, Giuliani D, Grassi T, Landoni F, Bonazzi CM, Fruscio R (2019). Ovarian function, fertility, and menopause occurrence after fertility-sparing surgery and chemotherapy for ovarian neoplasms. Gynecol Oncol.

[23] Gershenson DM, Miller AM, Champion VL, Monahan PO, Zhao Q, Cella D, Williams SD (2007). Gynecologic oncology group. Reproductive and sexual function after platinum-based chemotherapy in long-term ovarian germ cell tumor survivors: a gynecologic oncology group study. J ClinOncol..

[24] Matei D, Miller AM, Monahan P, Gershenson D, Zhao Q, Cella D, Champion VL, Williams SD (2009). Chronic physical effects and health care utilization in long-term ovarian germ cell tumor survivors: a gynecologic oncology group study. J ClinOncol..

[25] Delanoy N, Pécuchet N, Fabre E, Combe P, Juvin K, Pujade-Lauraine E, OudardS (2015). Bleomycin-induced pneumonitis in the treatment of ovarian sex cord-stromal tumors: a systematic review and Meta-analysis. Int J Gynecol Cancer.

[26] Skalleberg J, Solheim O, Fosså SD, Småstuen MC, Osnes T, Gundersen PO, Bunne M (2017). Long-term ototoxicity in women after cisplatin treatment for ovarian germ cell cancer. Gynecol Oncol.

[27] Chiannilkulchai N, Pautier P, Genestie C, Bats AS, Vacher-Lavenu MC, Devouassoux-Shisheboran M, Treilleux I, Floquet A, Croce S, Ferron G, Mery E, Pomel C, Penault-Llorca F, Lefeuvre-Plesse C, Henno S, Leblanc E, Lemaire AS, Averous G, Kurtz JE, Ray-Coquard I (2017). Networking for ovarian rare tumors: a significant breakthrough improving disease management. Ann Oncol.

[28] Joly F, Héron JF, Kalusinski L, Bottet P, Brune D, Allouache N, Macé-Lesec'h J, Couëtte JE, Pény J, Henry-Amar M (2002). Quality of life in long-term survivors of testicular cancer: a population-based case-control study. J ClinOncol.

[29] Smets EM, Garssen B, Bonke B, De Haes JC (1995). The multidimensional fatigue inventory (MFI) psychometric qualities of an instrument to assess fatigue. J Psychosom Res.

[30] Gentile S, Delarozière JC, Favre F, Sambuc R, San Marco JL (2003). Validation of the French “multidimensional fatigue inventory” (MFI-20). Eur J Cancer Care.

[31] Gernier F, Joly F, Klein D, Mercier M, Velten M, Licaj I (2020). Cancer-related fatigue among long-term survivors of breast, cervical, 486 and colorectal cancer: a French registry-based controlled study. Support Care Cancer.

[32] Cella DF, Tulsky DS, Gray G, Sarafian B, Linn E, Bonomi A, Silberman M, Yellen SB, Winicour P, Brannon J (1993). The functional assessment of Cancer therapy scale: development and validation of the 476 general measure. J Clin Oncol.

[33] Zigmond AS, Snaith RP (1983). The hospital anxiety and depression scale. Acta Psychiatr Scand.

[34] Joly F, Lange M, Rigal O, Correia H, Giffard B, Beaumont JL, Clisant S, Wagner L (2012). French version of the functional assessment of Cancer therapy-cognitive function (FACT-cog) version 3. Support Care Cancer.

[35] Bastien CH, Vallières A, Morin CM (2001). Validation of the Insomnia Severity Index as an outcome measure for insomnia research. Sleep Med.

[36] Andrade LF, Ludwig K, Goni JMR, Oppe M, de Pouvourville G (2020). A French value set for the EQ-5D-5L. Pharmacoeconomics..

[37] Margier J, Baffert S, Le Corroller-Soriano AG (2018). French Costing Group. Standard or Specific Unit Costs: Which Criteria for Choosing an Economic Evaluation of Health Strategies in Multicentric Studies?. Rev Epidemiol Sante Publique.

[38] Strumberg D, Brügge S, Korn MW, Koeppen S, Ranft J, Scheiber G, Reiners C, Möckel C, Seeber S, Scheulen ME (2002). Evaluation of long-term toxicity in patients after cisplatin-based chemotherapy for non-seminomatous testicular cancer. Ann Oncol.

